# Effects of a digital psychosocial intervention on somatic complaints, sleep latency problems, and body size perceptions in youth after parental separation: A randomized controlled trial

**DOI:** 10.1371/journal.pdig.0001460

**Published:** 2026-06-18

**Authors:** Andreas Nielsen Hald, Daniel Bach Johnsen, Gert Martin Hald, Camilla Stine Øverup

**Affiliations:** 1 Department of Public Health, Aarhus University, Aarhus C, Denmark; 2 Department of Public Health, University of Copenhagen, København K, Denmark; University of Pittsburgh School of Medicine, UNITED STATES OF AMERICA

## Abstract

Parental relationship dissolution is a disruptive event in children and adolescents’ lives that can negatively affect mental health, psychosocial adjustment, and everyday well-being. Psychosocial interventions are typically evaluated using measures of mental health and psychosocial adjustment, but less is known about whether they also improve less obvious well-being indicators such as somatic complaints, sleep latency problems, and body size perceptions. This study examined the effects of SES NXT, a digital psychosocial intervention from the Samarbejde Efter Skilsmisse (SES; “Cooperation After Divorce”) platform, on these outcomes among children and adolescents (ages 3–17; referred to collectively as youth) experiencing parental relationship dissolution. We conducted a randomized controlled, parallel-group, superiority trial comparing youth who received access to SES NXT with those in a waitlist control group. The sample included 467 families and 866 youth recruited across Denmark. Data were collected at baseline, 4 weeks, and 12 weeks after enrollment. Outcomes were assessed with single self- or parent-reported items on somatic complaints, sleep latency problems, and perceived body size, drawn from previous research. Multilevel regression models using generalized estimating equations tested group differences at the 12-week endpoint, adjusting for baseline scores and demographic covariates. At 12 weeks, youth in the waitlist control group had higher odds of reporting somatic complaints (*OR* = 4.18), sleep latency problems (*OR* = 3.99), and extreme body size perceptions (*OR* = 2.59) than youth in the intervention group. Differences in somatic complaints and sleep latency problems were evident at 4 weeks and remained through 12 weeks. These findings suggest that a digital psychosocial intervention may also affect less obvious self- or parent-reported well-being indicators among youth following parental relationship dissolution.

## Author summary

When parents split up, it can be stressful for children and affect their day-to-day well-being. Support programs are often judged by whether they reduce obvious signs of distress, such as sadness or anxiety. But children may also show stress in less direct ways, like headaches or stomach aches, trouble falling asleep, or feeling unhappy with their body size. We know little about whether online support programs improve these less obvious signs of well-being. In this study, we tested a newly developed online program for children and adolescents aged 3–17. It uses age-appropriate stories and exercises to support coping and communication. We recruited 467 families with 866 children in Denmark. Half received access to the program right away, while the other half were placed on a waitlist. We asked families about headaches and stomach aches, problems falling asleep, and body size perceptions at the start, after 4 weeks, and after 12 weeks. After 12 weeks, children in the program group reported fewer complaints, fewer problems falling asleep, and fewer extreme body size perceptions than children in the waitlist group. This suggests that online support may promote youths’ well-being more broadly after parental separation.

## Introduction

Parental relationship dissolution is a common and significant stressor in children’s lives, introducing various changes to family dynamics such as disruptions in routines, new living situations and family structures, and economic instability [1]. These stressors may have both immediate and long-term effects. In the short term, children may show acute distress, concentration problems, and dips in school adjustment [1,2]. Longer-term, some can experience lower educational attainment and be at greater risk of relationship instability, including divorce in adulthood [1,2]. Consistent with this, various studies have shown that parental divorce is associated with children’s poorer mental and emotional well-being, such as higher levels of depression [3–5], as well as reduced psychological and social functioning [1,2,6].

Although much attention has been given to the mental and social consequences for children of parental relationship dissolution, research also suggests that it may be associated with other, less obvious aspects of well-being [7–12]. Children who experience parental relationship dissolution may, for example, be at heightened risk for outcomes such as chronic daily headaches [12], disrupted sleep patterns [13], and body dissatisfaction [14]. These less obvious aspects of well-being may reflect stress and psychosocial strain associated with parental relationship dissolution and may also constitute important indicators of well-being in their own right [12–15].

Given these multifaceted psychosocial and well-being challenges associated with parental relationship dissolution, there is a growing need for interventions that support children through this transition. Several in-person, school-based group interventions have been developed to help children navigate the psychological consequences of parental separation [16–18]. These interventions are typically delivered in group formats by a coach or therapist and have shown promise in reducing internalizing and externalizing problems [19–21]. However, they can be resource-intensive and often have limited reach and scalability, particularly in rural or underserved areas [22].

Digital health interventions present a scalable and accessible alternative. Digital interventions can offer emotional and behavioral support in ways that are cost-effective, engaging, and not limited by geography [20,22–27]. Prior studies have shown that digital interventions can improve both short-term and long-term outcomes among adults experiencing relationship dissolution [23,28–30]. For children, digital interventions such as Children of Divorce – Coping with Divorce (CoD-CoD) have demonstrated effectiveness in reducing anxiety and depression symptoms [24].

Despite these promising developments, digital psychosocial interventions for youth experiencing parental relationship dissolution have mainly been evaluated using measures of mental health and adjustment. Less is therefore known about whether such interventions also influence the less obvious well-being indicators that may follow parental separation. This gap matters because children’s psychosocial environments are closely linked to their everyday well-being and functioning. For example, children in nuclear families report better health-related quality of life than those in joint or single-custody arrangements [8], and exposure to family conflict and instability following separation has been associated with poorer self-rated health in young adulthood [31].

To address this gap, we investigate SES NXT, a digital intervention that is age-adapted and includes interactive content such as videos, stories, and exercises that target key psychosocial domains including emotion regulation, coping strategies, and parent–child communication. SES NXT was developed to address the limited availability of scalable, age-adapted digital interventions for youth experiencing parental relationship dissolution and spans a broad developmental range from early childhood to late adolescence among 3- to 17-year-olds [32].

The current study is part of a larger pre-registered randomized controlled trial evaluating multiple outcome domains of the SES NXT intervention [32]. Whereas the primary trial outcomes focus on emotional and behavioral adjustment [33], the current study examines self- or parent-reported well-being indicators commonly reported during parental relationship dissolution: somatic complaints, sleep latency problems, and body size perceptions [7,10,12,13,34–36]. These outcomes represent a conceptually distinct domain and address a separate digital health question: whether psychosocial digital interventions may also influence less obvious complaints and perceptions that are not directly targeted by intervention content.

Taken together, we hypothesize that, at the 12-week follow-up, youth in the intervention group will report fewer somatic complaints (i.e., headaches, abdominal pain, nausea; H1), fewer days with problems with sleep latency (H2), and fewer extreme body size perceptions (H3) compared to the waitlist (WL) control group.

## Results

**Fig 1:**
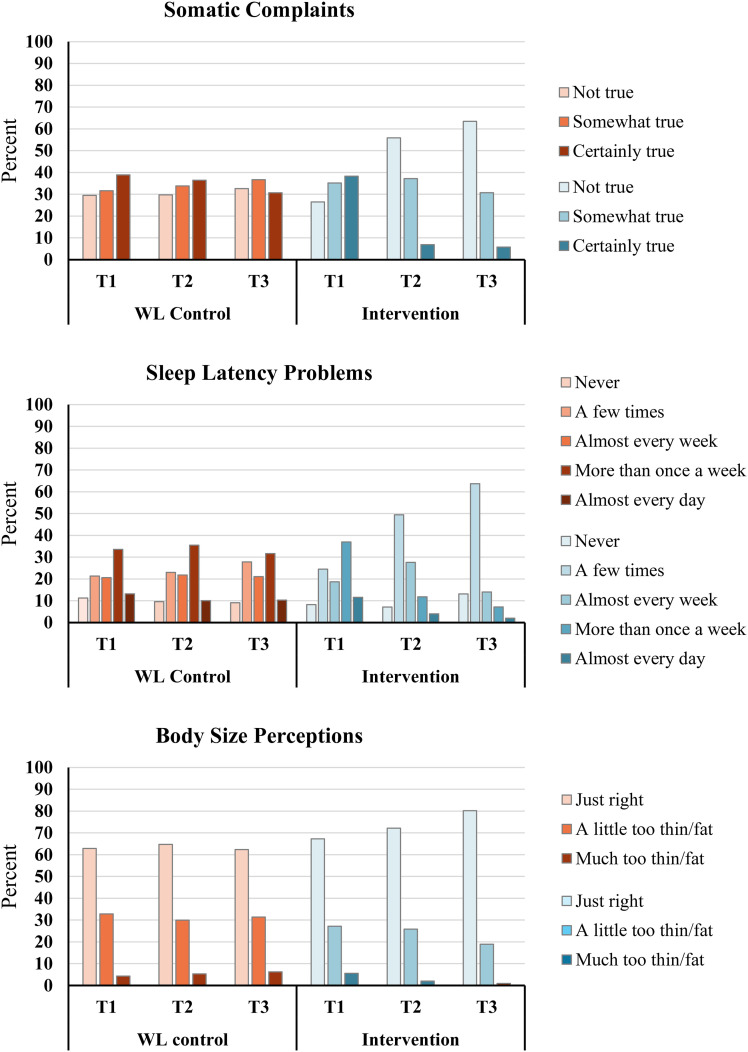
Distribution of responses for somatic complaints, sleep latency problems, and body size perceptions across study groups and time points. Bars show the percentage distribution of response categories for the WL control and intervention groups at baseline (T1), 4 weeks after enrollment (T2), and 12 weeks after enrollment (T3).

Fig 1 depicts the distribution of responses for somatic complaints, body size perceptions, and sleep latency problems. For all three outcomes, the distribution of responses remains relatively stable across time (at baseline (T1), 4 weeks after enrollment (T2), and 12 weeks after enrollment (T3)) for the control group, while for the intervention group, the responses shift, such that more responses are provided at the lower end of the response scale (e.g., “not true” for somatic complaints, “a few times” for sleep latency problems, and “just right” for body size perceptions).

Table 1 presents the results of the group comparisons at T3 for the three outcomes. At T3, youth in the WL control group had significantly higher odds of reporting somatic complaints (odds ratio *[OR]* = 4.18), sleep latency problems (*OR* = 3.99), and more extreme body size perceptions (i.e., too thin/fat; *OR* = 2.59) than youth in the intervention group.

**Table 1:** Tests of effectiveness of the intervention, as defined by a test of group differences at T3.

Outcome	Estimate	St. Error	*95% CI*		*z*	*p*	*OR*
Somatic complaints	1.43	0.16	[1.11;	1.75]	8.80	<.001	4.18
Sleep latency problems	1.38	0.17	[1.04;	1.72]	7.97	<.001	3.99
Body size perceptions	0.95	0.22	[0.52;	1.39]	4.31	<.001	2.59

**Note**. N_intervention_ = 449, N_WL_ = 417. T3 = 12-week follow-up. Group membership is coded 0 = WL control group and 1 = intervention group; the intervention group is the reference group for the comparisons. The results for the between-group difference come from Analysis of Generalized Estimating Equation (GEE) Parameter Estimates and the *lsmeans* statement, which provide standard output from SAS *proc genmod*. Covariates were included in the analysis.

Table 2 presents the marginal, covariate-adjusted predicted probabilities. For somatic complaints, the predicted probability of reporting symptoms as “certainly true” was higher in the WL control group than in the intervention group (0.24 vs. 0.07), as was the predicted probability of reporting symptoms as “somewhat true” (0.69 vs. 0.35). A similar pattern was observed for sleep latency problems, with higher predicted probabilities of more frequent sleep difficulties in the WL control group, including difficulties occurring “almost every day” (0.06 vs. 0.02) and “more than once per week” (0.32 vs. 0.10). For body size perceptions, the predicted probability of reporting “a bit too thin or too fat” was higher in the WL control group than in the intervention group (0.29 vs. 0.13), whereas the predicted probability of reporting “too thin or too fat” was near zero in both groups. Taken together, these results indicate that, at T3, youth in the intervention group had substantially lower odds of reporting somatic complaints, sleep latency problems, and more extreme body size perceptions than youth in the WL control group, corresponding to approximately 76%, 75%, and 61% lower odds, respectively.

**Table 2:** Marginal, covariate-adjusted predicted probabilities of responses to the three outcomes at T3, for intervention and WL control group separately.

	Intervention group	WL Control Group
Somatic Complaints		
Certainly true	0.07	0.24
Somewhat true	0.35	0.69
Sleep Latency Problems		
Almost every day	0.02	0.06
More than once per week	0.10	0.32
Almost every week	0.26	0.58
A couple of times	0.87	0.96
Extreme Body Size Perceptions		
Too thin/fat	0.01	0.01
A bit too thin/fat	0.13	0.29

Note. N_intervention_ = 449, N_WL_ = 417. Values represent covariate-adjusted predicted probabilities for each response category at T3 estimated from the GEE models. SAS does not provide probabilities for the reference categories (“Not true”, “Never”, and “Just right”).

This was further supported by analyses examining whether group differences in the odds of reporting somatic complaints, sleep latency problems, and more extreme body size perceptions changed over time. Table 3 shows that the Group × Time interaction was statistically significant for all three outcomes. Table 4 presents group comparisons at each time point and shows that the groups did not differ at T1. However, group differences were evident at later time points and were larger at T3 than at T2.

**Table 3:** Tests of effectiveness of the intervention, as defined by a test of group differences over time, as well as tests of the effect of intervention age, version and intervention use.

Outcome	Group × Time		Intervention age group^a^		Modules^a^	
	*Chi-square*	*p-value*	*Chi-square*	*p-value*	*Chi-square*	*p-value*
Somatic complaints	78.93	<.001	4.36	0.225	0.34	0.558
Sleep latency problems	73.39	<.001	0.15	0.985	1.70	0.192
Body size perceptions	20.10	<.001	3.12	0.374	7.57	0.006

**Note**. N_intervention_ = 449, N_WL_ = 417. Ns for each intervention age group were 3–5 years = 109, 6–8 years = 115, 9–12 years = 122, and 13–17 years = 103. ^a^ indicates that only the intervention group was included in the analysis. The test statistics (chi-square and p-value) come from a Type 3 Generalized Estimating Equation (GEE) Analysis. These tests are standard output from SAS proc genmod; model/ type3.

**Table 4:** Tests of comparison between the WL control group and intervention group at each time point for somatic complaints, sleep latency problems, and body size perceptions.

			Somatic complaints			Sleep latency problems			Body size perceptions		
Comparison			Estimate	*p*	*OR*	Estimate	*p*	*OR*	Estimate	*p*	*OR*
Control group vs.	Intervention group	at T1	-0.05	0.728	0.95	-0.14	0.306	0.87	0.17	0.259	1.18
Control group vs.	Intervention group	at T2	1.28	<.001	3.60	0.75	<.001	2.11	0.28	0.061	1.33
Control group vs.	Intervention group	at T3	1.33	<.001	3.76	1.21	<.001	3.35	0.84	<.001	2.31

**Note**. N_intervention_ = 449, N_WL_ = 417. Group membership is coded 0 = WL control group and 1 = intervention group; intervention group is used as the reference group in comparisons. The differences were estimated using an LSMEANS statement (in SAS *proc genmod*). Covariates were included in the analysis.

The supplemental materials provide estimated probabilities for responses to the three outcomes across time points (Tables C, D, and E in S1 Appendix). These tables show how the predicted probabilities changed over time in the intervention and WL control groups. For somatic complaints, the predicted probability of reporting symptoms as “certainly true” decreased substantially in the intervention group from T1 (34.2%) to T3 (8.6%), whereas it remained relatively stable in the WL control group (33.1% at T1 and 26.1% at T3). A similar pattern was observed for the “somewhat true” response category. For sleep latency problems, the predicted probabilities also declined more in the intervention group than in the WL control group between T1 and T3. For example, for the “almost every day” response category, the predicted probability decreased from 10.7% at T1 to 2.4% at T3 in the intervention group, compared with a change from 9.5% to 7.5% in the WL control group.

For body size perceptions, the predicted probability of responding “a little too thin” or “a little too fat” declined in the intervention group from T1 (32.2%) to T3 (19.8%) but remained relatively stable in the WL control group (35.9% at T1 and 36.3% at T3). A similar pattern was observed for the more extreme body size perception categories (“much too thin” or “much too fat”).

There was no significant difference in outcome scores between the different age versions of the intervention, indicating no evidence that intervention effects differed across the four age versions (see Table 3). However, because parent-report was used for younger children and self-report for older youth, age-related comparisons should nevertheless be interpreted with caution as they may also reflect differences in informant type.

Approximately 80% of youths in the intervention group accessed the SES NXT intervention. Most youths (~72%) accessed the first module on the same day as or the day after completion of the T1 survey, and most module engagement occurred within 1 day (~93%). Tables showing the number of modules completed and the frequency of completion of specific modules are provided in the supplemental materials (Tables F and G in S1 Appendix). A dose-response association was observed only for body size perceptions, such that greater module engagement was associated with lower odds of reporting more extreme body size perceptions (*b* = -0.19, *S.E.* = 0.07, 95% CI [-0.33; -0.05], *z* = -2.66, *p* < .008, *OR* =.83). This association did not, however, persist in sensitivity analyses using the unimputed data (see Fig A and Tables K–S in S1 Appendix). Follow-up analyses examined whether engagement with specific intervention themes (see Table 6 in the Methods section) was associated with the outcomes. For body size perceptions, greater engagement with modules in the emotional aspects of parental divorce theme was associated with lower odds of reporting more extreme body size perceptions (*b* = -0.48, *S.E.* = 0.23, 95% CI [-0.92; -0.03], *z* = -2.09, *p* = .036, *OR* =.62) (see Table H in S1 Appendix). Because the platform recorded whether modules were started or completed, but not time spent, attention, or depth of processing, these engagement measures should be interpreted as rough indicators of intervention use.

**Table 6:** Intervention content overview, by age group in the intervention.

Theme	3-5 years	6-8 years	9-12 years	13-17 years
**Family Constellations**				
My Family	X			
The Bonus Family		X	X	X
**Practical Matters**				
Changeover Day	X			
Living in Two Places		X	X	X
Packing Your Bag		X	X	X
**Emotional Aspects of Parental Divorce**				
Missing Someone Means You Care	X			
Feelings	X	X		
Understand Your Feelings			X	X
When It has Just Happened		X	X	X
Tell Your Story			X	X
**Agency**				
Find an Important Adult		X	X	X
Learn To Say Yes and No		X	X	X
My Parents Are Not Getting Along		X	X	X
My Rights			X	X

## Discussion

### Principal findings

This RCT examined the effects of the SES NXT digital intervention on self- or parent-reported well-being indicators in youth experiencing parental relationship dissolution. At 12 weeks (T3), youth in the intervention group had lower odds of reporting somatic complaints, sleep latency problems, and extreme body size perceptions than youth in the control group. Specifically, the intervention group had approximately 76% lower odds of reporting somatic complaints such as headaches, stomachaches, or nausea, 75% lower odds of reporting sleep latency problems, and 61% lower odds of reporting extreme body size perceptions. Group differences in somatic complaints and sleep latency problems were already evident at 4 weeks (T2) and remained evident at 12 weeks (T3).

### Comparison to prior work

The observed effects should be considered in relation to both the broader SES NXT trial and prior intervention research. In the companion paper from the same trial, the primary mental health outcomes at 12 weeks were in the medium to large range (Cohen’s *d* = .66 to.71), and the secondary mental health outcomes were in the medium range (Cohen’s *d* = .47 to.56) [33]. Although these Cohen’s *d* values are not directly comparable to the odds ratios reported in the present study, they indicate that the current findings are consistent with a broader pattern of improvements across outcomes within the trial.

Relative to prior research in this and adjacent areas outside the trial, however, the present estimates appear comparatively large. In the most directly comparable digital intervention for youth after parental divorce, CoD-CoD, the intervention improved self-reported emotional problems with an effect size of Cohen’s *d* = 0.37 and reduced the rate of clinically significant self-reported mental health problems at 1 month with *OR* = 0.58 [24]. More broadly, a meta-analysis of postdivorce interventions for children found no significant overall effects on children’s mental health outcomes and only small, non-significant pooled effects for internalizing symptoms, depression, anxiety, and combined anxiety/depression, whereas stronger effects were observed for more proximal outcomes such as self-esteem (Hedges’ *g* = 0.56) and divorce adjustment (Hedges’ *g* = 0.70) [37]. Similarly, a meta-analysis of internet-delivered interventions for children and young people with depression and anxiety symptoms found a small effect for anxiety symptoms (Hedges’ *g* = -0.25), a small but non-significant effect for depression (Hedges’ *g* = -0.27), a moderate effect for impaired functioning (Hedges’ *g* = 0.52), and no effect on quality of life (Hedges’ *g* = -0.01) [38]. Taken together, these comparisons suggest that the observed effect sizes are large relative to previous research.

### Interpretation

As discussed above, the observed effects appear comparatively large relative to prior intervention research and should therefore be interpreted with some caution. Specifically, the trial may have provided a particularly favorable context for detecting such effects; we consider three key features of the study that may help explain why.

First, SES NXT was tested in Denmark, a high-income country with universal access to healthcare, a strong welfare system, and high digital literacy among both adults and children [39]. Health and welfare services are decentralized and organized through shared responsibilities across the state, regions, and municipalities [40]. Denmark is also highly ranked on the Digital Government Index, and most citizens routinely interact with public institutions through online platforms [41]. The Danish context is further characterized by relatively low stigma surrounding divorce and a formal support infrastructure for families going through separation [15]. The Danish case may thus have created a particularly favorable context for testing a digital intervention such as SES NXT, which may have contributed to the observed effects through, for example, greater awareness, easier recruitment, stronger perceived legitimacy, and higher uptake. The use of SES NXT in the present setting may therefore differ from what would be observed in countries with lower levels of digital literacy, support, and access or where public trust in digital interventions is less established.

Second, the sample was self-selected and youth enrolled at baseline showed elevated levels of somatic complaints, sleep latency problems, and extreme body size perceptions. This may suggest that participating families were not fully representative of all families experiencing divorce, but rather included families for whom relationship dissolution was associated with greater difficulties, or who had a stronger perceived need for additional support. Such selectivity may have increased the room for improvement and could therefore have contributed to the magnitude of the observed effects. At the same time, this does not undermine the internal comparison between the intervention and waitlist control groups, as participants were randomized and the groups did not differ meaningfully at baseline. Moreover, the sample is highly relevant to the intended use of the intervention as SES NXT is designed to support families and youth who experience difficulties after relationship dissolution. Recruitment through municipalities and the Agency of Family Law is therefore a strength, as these are the channels through which the intervention is implemented and through which the intended target population would be expected to encounter the intervention. Thus, while self-selection should be considered when interpreting the size and generalisability of the effects, the sample remains appropriate for evaluating the intervention among those it is designed to reach.

Third, the outcome measures themselves may also have contributed to the effect sizes. All three outcomes were assessed using single self- or parent-reported items. Although such items may capture meaningful change, single items capture less of the construct as compared to multi-item scales and may be more sensitive to random measurement error. In an unblinded waitlist trial, some of the observed between-group differences may also reflect expectancy or reporting effects, whereby youth or parents in the intervention group interpreted or reported these experiences more positively over time. In a selected sample with relatively high baseline symptom levels, these features may therefore also have contributed to the size of the observed effects.

Taken together, the findings indicate large observed effects in a setting that may approximate a best-case scenario for uptake and response and may therefore have been amplified by features of the sample and the measurement strategy. The observed effects could thus be understood as reflecting a combination of intervention effects, implementation context, and measurement conditions. Against this background, several possible pathways may help explain the intervention’s contribution to the observed findings, noting that these mechanisms were not directly examined in the present analyses.

Somatic complaints, the experience of physical symptoms that may be linked to emotional distress, are especially common in children experiencing parental relationship dissolution [7,10,12,15]. Symptoms such as headaches, abdominal pain, and nausea frequently occur without any identifiable medical cause and often serve as a means through which children express emotions they are unable to verbalize [7,12]. Younger children, in particular, may struggle to articulate complex feelings such as fear, sadness, or confusion and may instead manifest these emotions physically [12]. One possible interpretation of the present findings is therefore that SES NXT may have helped some youth better identify and regulate emotions, feel more understood, and adopt more adaptive coping strategies, which in turn may have reduced the reporting of somatic complaints.

Difficulty with sleep is also a common concern among youth who have experienced a disruption in their family structure [13,36]. Changes relating to bedtime routines and having to sleep in new places can interfere with sleep quality and duration [36]. Youth in these situations frequently report trouble falling asleep, and chronic activation of the stress response may further impair the body’s natural regulatory systems, leading to hyperarousal and bedtime anxiety [13,36]. Although SES NXT did not directly target sleep hygiene, its broader focus on emotional safety, predictability, and self-regulation, including practical themes related to living across two households, may have reduced bedtime anxiety and rumination and thereby contributed to lower sleep latency problems.

Lastly, extreme body size perceptions are increasingly recognized as an important health-related self-perception in children and adolescents [9,34,35,42]. This self-perception can be intensified during parental relationship dissolution because of changed eating habits, inconsistent meal routines, and reduced parental oversight [42]. Such circumstances can alter youths’ relationships with their bodies, particularly if food becomes a source of comfort or conflict [34]. Furthermore, youth experiencing family instability may internalize negative self-perceptions that extend to how they view their physical appearance [35]. A possible interpretation is therefore that SES NXT, by supporting self-understanding, emotional resilience, and expression of difficult feelings, may have indirectly influenced how some youth viewed their bodies.

### Limitations

Several limitations should be considered when interpreting the results of this study.

First, because SES NXT was evaluated under conditions that may have been especially favorable for uptake and response, the size of the observed effects may differ across other populations or contexts.

Second, all outcomes were assessed using single self- or parent-reported items. Single items may not capture the entire construct of interest and may be more sensitive to random measurement error, reporting effects (e.g., recall bias, social desirability bias), and shifts in how complaints are interpreted over time. The findings should be interpreted with these measurement limitations in mind.

Third, outcomes were reported by different informants depending on the child’s age. Parents completed questionnaires for children aged 3–10 years, whereas youth aged 11–17 years were asked to complete the questionnaires themselves. Because the surveys were administered online, we cannot fully verify whether adolescents completed them independently or with parental assistance. As a result, concordance between parent- and self-reports cannot be assessed, and it is not possible to disentangle whether age-related differences reflect developmental differences in symptoms or differences between informants. In the analyses, age was associated with higher odds of reporting somatic complaints (see results output: https://osf.io/5bpva/). However, because informant type varies with age in the present design, it remains unclear whether this pattern reflects higher symptom levels among older youth or potential underreporting by parents of younger children [43].

Fourth, we changed the imputation strategy from multiple imputation to single imputation which may have resulted in smaller estimated variances and a higher risk of Type I error. To assess the possible implications of missing data, we added descriptive missingness information and attrition analyses, conducted the GEE analyses on the unimputed data, and explored likelihood-based ordinal models. The analyses using the unimputed data showed the same overall pattern for the main group comparisons. The likelihood-based mixed-effects ordinal models did not converge with the full nested structure, but simpler fixed-effects likelihood-based models yielded results that were substantively similar in pattern to the reported GEE models. Hence, although missing data remains a methodological limitation, the available sensitivity analyses did not suggest that the main group comparisons depended on the imputation strategy.

Fifth, the 12-week follow-up period may be a limitation as it does not allow for conclusions about the long-term sustainability of the observed effects. The duration of the waitlist control period was determined by both ethical and pragmatic considerations. Because the intervention targeted children and adolescents experiencing parental relationship dissolution, a potentially vulnerable group, prolonged withholding of access was considered ethically problematic by funding bodies and collaborating municipalities. Extending the waitlist period was also expected to increase attrition in the control group, which could have threatened the trial’s internal validity. The 12-week endpoint therefore reflected a balance between allowing time for intervention effects to emerge and limiting ethical concerns and differential dropout.

Finally, although the digital format of SES NXT offers accessibility and scalability, it also introduces the possibility of technical barriers that were not systematically captured in this study. Issues such as unstable internet access, device limitations, or difficulties using the platform may have influenced the extent to which participants engaged with the intervention content. Such barriers would most likely have reduced or unevenly distributed exposure to the intervention, and may therefore have attenuated the observed effects.

## Methods

The procedure for the RCT is described in detail elsewhere [32]. We did not deviate from the procedures described therein, with one exception; we changed the missing data imputation strategy to single imputation, as the amount of missing data was relatively low (roughly 12%). The CONSORT checklist is provided as Table T in S1 Appendix. These data come from a larger study that assessed multiple constructs; results for the primary outcome (mental health and well-being) are reported in [33], and results for an exploratory outcome (divorce conflict) are reported in [44].

### Sample size

We simulated the data structure and assumed moments (mean difference and outcome pooled standard deviation) to determine the minimum sample size for this study using R, version 4.4.1. The sample size was calculated with reference to the primary study hypothesis (the Strengths and Difficulties Questionnaire (SDQ) emotional subscale score; reported elsewhere), assuming a power of 90% and an alpha level of.05. We expected a small to moderate effect, as evidenced by a raw mean difference of.40 in the SDQ emotional subscale score between the intervention and control group. Power was determined for a cross-sectional multi-level model, with children nested within parent, using generalized estimating equation (GEE). The simulation suggested that roughly 700 clusters (parents with children) would be needed.

### Eligibility criteria and participants

To be eligible to participate, parents had to have gone or currently go through relationship dissolution, have at least one custodial child between the ages of 3 and 17 at study inclusion, and access to an electronic device (phone, tablet, or computer) with access to the internet. Parents, and their children, must be able to read and understand Danish, as all materials were provided in Danish only.

**Fig 2:**
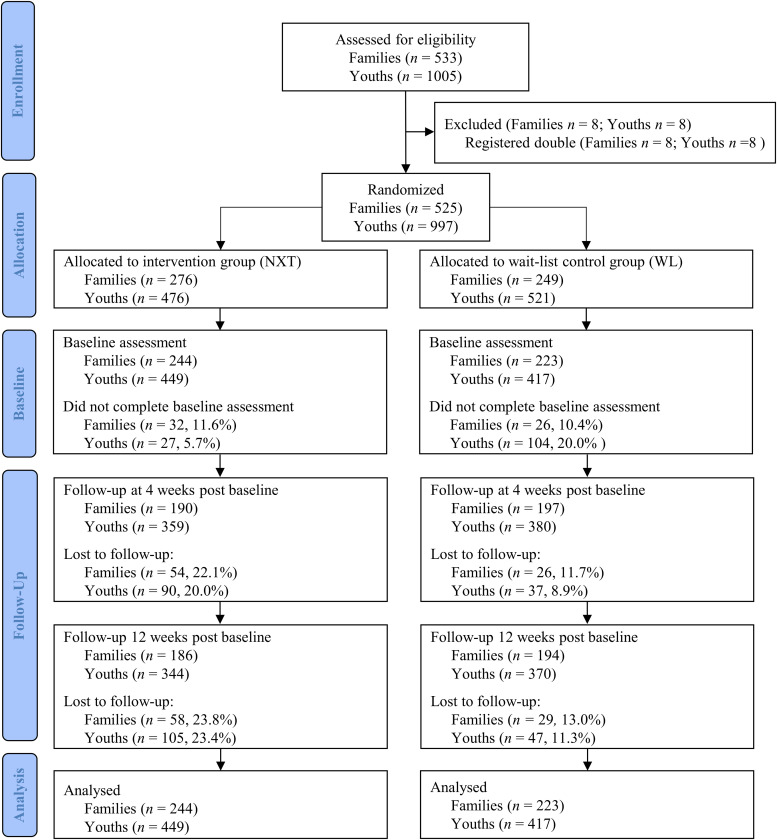
CONSORT diagram of participant flow through the trial. The diagram shows the number of families and youth assessed for eligibility, randomized, allocated to the SES NXT intervention or waitlist (WL) control group, followed up at 4 weeks (T2) and 12 weeks (T3), and included in the intention-to-treat analyses. Attrition rates at follow-up were calculated with reference to the baseline sample size (T1).

Participants were recruited between May 1st, 2023, and August 31st, 2024, and data collection ended on January 31^st^, 2025. A total of 533 families and 1,005 youths were assessed for eligibility. Sixty-six families (139 youth) were excluded due to double registration or failure to provide baseline data; thus, the final sample, based on the intention-to-treat (ITT) principle, consisted of 467 families and 866 youth (see Fig 2 for the CONSORT diagram).

As evident in the CONSORT diagram, there was differential attrition from the study for the intervention and the WL control group. Attrition analyses showed that being in the WL group, reporting more sleep latency problems at baseline, and having higher parental income were associated with lower odds of dropping out, whereas older parental age was associated with higher odds of dropping out (Table I in S1 Appendix).

Table 5 provides a breakdown of the demographic make-up of the sample. Most of the parents signing the family up for participation were the biological mother, signing up two youth, and the ex-partner was almost exclusively the youth’s other biological parent. On average, the relationship had lasted 10.9 years and ended 2.0 years prior to study enrollment. About half of parent participants indicated that they were the divorce initiators and most parent participants indicated that either they and/or their partner had new partners. The average age of parent participants was 40.2 years, and most had below-average income and had a low educational level.

**Table 5:** Baseline characteristics of participants in the Intention-To-Treat (ITT) population.

Characteristic	Study group^a^		
Demographic, Youth	NXT (*n =* 449)	WL (*n* = 417)	All (*N* = 866)
Girls	229 (51.0)	209 (50.1)	438 (50.6)
Age, mean (SD)	9.0 (4.1)	9.0 (4.3)	9.0 (4.2)
Survey Age group			
3-5	109 (24.3)	108 (25.9)	217 (25.1)
6-10	193 (43.0)	162 (38.8)	355 (41.0)
11-17	147 (32.7)	147 (35.3)	294 (33.9)
Intervention Age group			
3-5	109 (24.3)	108 (25.9)	217 (25.0)
6-8	115 (25.6)	109 (26.1)	224 (25.9)
9-12	122 (27.2)	96 (23.0)	218 (25.2)
13-17	103 (22.9)	104 (24.9)	207 (23.9)
Modules completed by intervention age group, mean (SD)	4.0 (2.4)		
3–5 ^b^	1.0 (0.0)	--	--
6-8	4.8 (1.8)	--	--
9-12	5.3 (2.2)	--	--
13-17	5.0 (1.6)	--	--
**Demographic, Parent**	**NXT (*n = 2*44)**	**WL (*n* = 223)**	**All (*N* = 467)**
Age, mean (SD)	*40.2 (6.0)*	*40.3 (6.4)*	*40.2 (6.2)*
Parent registered as informant			
Biological mother	158 (64.8)	147 (65.9)	305 (65.3)
Biological father	86 (35.3)	76 (34.1)	162 (34.7)
Ex-partner, in relation to child			
Biological mother	85 (34.8)	72 (32.3)	157 (33.6)
Biological father	157 (64.3)	144 (64.6)	301 (64.5)
Bonus parent/other	2 (0.8)	7 (3.1)	9 (1.9)
Region, Parent			
Capital Region of Denmark	84 (34.4)	80 (35.9)	164 (35.1)
Region Zealand	65 (26.6)	63 (28.3)	128 (27.4)
Region of Southern Denmark	31 (12.7)	33 (14.8)	64 (13.7)
Central Denmark Region	46 (18.9)	30 (13.5)	76 (16.3)
North Denmark Region	18 (7.4)	17 (7.6)	35 (7.5)
Parent highest educational level			
Low	112 (45.9)	109 (48.9)	221 (47.3)
Medium	73 (29.9)	70 (31.4)	143 (30.6)
High	59 (24.2)	44 (19.7)	103 (22.1)
Parent monthly income in DKK^c^			
Below average (<10.000-40.000)	103 (42.2)	110 (49.3)	213 (45.6)
Average (40.001-50.000)	68 (27.9)	47 (21.1)	115 (24.6)
Above average (50.001- > 80.000)	73 (30.0)	66 (29.6)	139 (29.7)
Relationship status^d^			
Divorced	133 (54.5)	111 (49.8)	244 (52.3)
Marital duration, mean (SD)	*11.0 (5.5)*	*10.8 (5.5)*	*10.9 (5.5)*
Time since divorce, mean (SD)	*1.9 (2.4)*	*2.1 (2.6)*	*2.0 (2.5)*
Split/break-up	94 (38.5)	97 (43.5)	191 (40.9)
Relationship duration, mean (SD)	*11.1 (4.6)*	*10.7 (4.6)*	*10.9 (4.6)*
Time since break-up, mean (SD)	*1.9 (1.8)*	*2.1 (3.0)*	*2.0 (2.5)*
Break-up initiator			
Predominantly me	120 (49.2)	110 (49.3)	230 (49.3)
Mutual	47 (19.3)	45 (20.2)	92 (19.7)
Predominantly ex-partner	77 (31.6)	68 (30.5)	145 (31.1)
New Partner			
Neither have new partners	96 (39.3)	91 (40.8)	187 (40.4)
Participant does, former spouse does not	55 (22.5)	49 (22.0)	104 (22.3)
Participant does not, former spouse does	48 (19.7)	51 (22.9)	99 (21.2)
Both have new partners	45 (18.4)	32 (14.4)	77 (16.5)
No. of youths signed up per family			
1	52 (21.3)	54 (24.2)	106 (22.7)
2	179 (73.4)	147 (65.9)	326 (69.8)
3	13 (5.3)	19 (8.5)	32 (6.8)
4	--	3 (1.4)	3 (0.6)

^a^Unless otherwise indicated, data are expressed as number (percentage) of participants. Percentages have been rounded and may not total 100. Mean (SD) are presented in italics.

^b^The age group 3–5 year-olds accessed 4 themes, built as 1 module.

^c^The average monthly income in Denmark was 46.927 DKK in 2023.

^d^Some participants did not report their relationship status or provide dates for key events (e.g., start of relationship, marrying, separating, or divorcing), so percentages may not sum to 100 and sample sizes (N) vary. Missing data was not imputed. Time is shown in years.

The average age of youth participants was 9 years, and the participants were evenly split between boys and girls. Those in the intervention group who accessed SES NXT completed about four modules on average.

### Ethical approval and protocol registration

All procedures were in accordance with the ethical standards of the institutional and national research committee and with the 1964 Declaration of Helsinki and its later amendments or comparable ethical standards. We received ethical approval from the University of Copenhagen Research Ethics Committee for Science and Health (case number 504–0290/21–5000), and from the Danish Data Protection Agency (case number 514–0699/22–3000). The study was exempt from national ethical evaluations following the rules and regulations as set forth by the Scientific Ethical Committees of Denmark (i.e., national ethics approval was not required). The protocol was registered with clinicaltrials.gov prior to data collection (ClinicalTrials.gov Identifier: NCT05760820).

### Procedure

We conducted a randomized controlled, parallel-group, superiority trial that compared the digital SES NXT intervention with a WL control group. Participants completed surveys at baseline (at study enrollment; T1), 4 weeks post-baseline (T2), and 12 weeks post-baseline (T3); the primary endpoint was 12 weeks post-baseline (T3). Fig 2 presents the CONSORT diagram.

We partnered with 21 Danish municipalities across the country and the Danish Agency of Family Law to recruit participants. Specifically, municipal officials shared informational fliers through the school/daycare online parent portals, and physical posters and postcards were hung in high-traffic areas within the schools and daycares. Moreover, municipal and the Danish Agency of Family Law caseworkers could refer clients to the study. The informational materials directed interested parents to a website that provided information about the study structure, data protection and participant rights, and the study’s incentive structure (described below). They were also provided with contact information for the study responsible and for technical support.

From there, they were directed to a sign-up form. On the sign-up form, they indicated whether they were the custodial parent of one or more youth aged 3–17 years and provided the name, birthdate, and contact information for each youth. Parents could provide their own e-mail and/or phone number, or that of their children. Consent was collected at the end of the sign-up form, with a check box. Custodial parents consented to study participation on behalf of themselves and their children; only the custodial parents who signed the children up for the project provided consent. Given the online nature of the study, we were unable to obtain child assent. However, we advised the parents to talk to their child about study participation and the use of SES NXT.

Upon sign-up to the study, the families were randomized to either the intervention or the WL group. Randomization occurred at the family level, such that parents and youth were randomized to the same group, at a 1:1 allocation ratio. Randomization was performed electronically, with a random number generator and they were informed of their group allocation after completing the baseline (T1) survey. Intervention group participants received access to the intervention immediately. Access to SES NXT was provided to the specific youth, for whom the T1 survey was completed. User accounts were automatically generated for each youth that was signed up and the allocation e-mail directed participants on how to access the intervention. Participants in the WL control group received access to SES NXT after T3, regardless of whether they responded to the T2 and T3 surveys; access was granted 90 days after inclusion into the study. Neither participants nor study personnel were blind to group assignment.

After signing up, participants (i.e., parents and youth aged 11–17) were sent e-mails and/or text messages containing links to the online T1 survey; parents also received a link to a short survey about themselves. Parents received individual links to questionnaires for each child aged 3–10 years and adolescents aged 11–17 years received links to surveys for them to complete on their own. In cases where youth did not have their own e-mail address or mobile phone number, parents may have listed theirs and shared the survey with the youth. Upon completion of the baseline (T1) questionnaire, participants received follow-up questionnaires 4 weeks (T2) and 12 weeks post-baseline (T3). Participants received the T3 survey, regardless of whether they completed the T2 survey.

To optimize participant retention, we implemented two strategies: 1) Reminders to participate: All participants were sent reminder e-mails and/or text messages to complete the questionnaires two and five days after the initial (T1, T2, or T3) survey link was sent out. 2) Compensation for time: We provided two movie tickets (value 180 DKK/24 EUR) for active participation. Specifically, participants in the control group received one movie ticket for completing two surveys and one additional movie ticket for completing all three surveys. Participants in the intervention group received one movie ticket for completing two surveys and three SES NXT modules. They received an additional movie ticket for completing all three surveys and three modules on the intervention platform. The tickets were provided for each child individually.

### Intervention format and content

SES NXT is a digital intervention for children and youth aged 3–17 years, who experience parental relationship dissolution. The intervention was developed through collaboration between psychologists specializing in child and family dynamics and experienced digital developers. In addition, an advisory board composed of professionals from five municipalities and the Agency of Family Law, who are actively involved in casework with children and families experiencing divorce, provided advice and guidance during the development phase. The intervention includes various elements such as text, videos, motion graphics, and voice-overs; moreover, there are digital activities and exercises that aim to have the youth apply the material to their own situation. SES NXT is designed to be adaptive to the youth’s age. The intervention contains four age groups that correspond to the Danish daycare and school system: 3–5 years, 6–8 years, 9–12 years, and 13–17 years. The format of the intervention was adapted to reflect the cognitive, social, emotional, and digital competencies of each of those age groups, with varying amounts of text (and reading level), interactive elements, and visualizations.

The intervention addresses key factors associated with adjustment to parental relationship dissolution, such as perceived support [45], coping skills [46,47], child-parent relations and communication [46,48–50], and the interpretation and meaning made of the relationship dissolution (e.g., loss, gain, change, healing) [51,52]. Several principles guided the development of the modules: 1) that human behavior, emotions, and cognitions are situated in a social context [53]; 2) that parental relationship dissolution may represent a radical change and that youth should be supported in reconnecting with values, hopes, or dreams, either in their original form or adapted to new circumstances [54]; 3) that youth should experience, and believe in, their ability to influence the world around themselves and not only be the passive recipient of what happens around them [55]; 4) that parental relationship dissolution may represent an ‘appropriate disturbance’, meaning a disruption or challenge that is constructive and suitable within a specific context, and that experiencing such a ‘disturbance’ may lead to growth or change without causing harm or undue stress [56]; 5) that difficult feelings (e.g., feeling that they have failed or done something wrong) are common, and that they are not alone in those feelings. Normalizing these feelings by showing them they are not alone can make them less distressing and intrusive [55]; and 6) that the ability to understand mental states of oneself and others [57] is important in communicating with others and aiding in coping with parental relationship dissolution.

Table 6 provides an overview of the modules included in the intervention for each age group. Each module comprises a theme identified in the literature as central to youths experiencing parental relationship dissolution (e.g., living in two homes, bonus families, and parental conflict). Most of the content in the intervention is covered across the age groups, though in age-appropriate ways. The intervention could be accessed at any time during the trial by logging into the intervention website from a mobile smartphone, tablet, or computer. Given that divorce is a heterogeneous process, and the experience of divorce is different for each individual, participants could choose the modules that were most relevant to them. The modules could be completed in any order, as little or much as desired, and repeated as needed.

### Measures

All questions were administered at T1, T2, and T3; parents completed questionnaires on behalf of their children aged 3–10, while youth aged 11–17 completed the questionnaires themselves.

#### General demographic questions.

Parents reported their *gender* (0 = male, 1 = female) and age (entered as a numeric value). They also reported on their highest level of *education*; educational level was transformed into three categories: 1 = low level of education (e.g., primary school, high school, business high school, vocational education), 2 = medium level of education (e.g., medium-cycle tertiary education, bachelor’s degree), and 3 = high level of education (e.g., master’s degree or higher). And they reported on their *monthly income* on a nine-point scale with 10,000 DKK intervals (app. 1,500 USD intervals), from 0 = “below 10,000 DKK” (i.e., below 1,500 USD) to 8 = “more than 80,000 DKK” (i.e., approximately 12,000 USD). Parents also provided information on their *past and current relationship status*, including whether they were legally divorced or had split/broken up if not legally married, the duration of this relationship, time since divorce, who initiated the break-up (i.e., “more me,” “mutual,” or “more the other”), and whether they or their former partner had entered a new relationship.

During the baseline survey, parents reported on their *child’s gender* (0 = male, 1 = female; youth aged 11–17 reported on their own gender), while *child age* was provided during the sign-up form (in the form of a birthdate that was converted to an age in whole years).

#### Parental mental health (Covariates).

Parental depression and anxiety symptoms, representing mental health, were assessed with the 2-item Patient Health Questionnaire (PHQ) (Little interest or pleasure in doing things; Feeling down, depressed, or hopeless; [58];.70/.51/.60, for each time point), and the 2-item Generalized Anxiety Disorder scale (GAD) (Feeling nervous, anxious or on edge; Not being able to stop or control worrying; [59];.78/.64/.75, for each time point). Participants were asked how frequently within the last 2 weeks they had experienced each of the symptoms and provided responses using the following scale: “Not at all” (0), “Several days” (1), “More than half the days” (2), or “Nearly every day” (3). Higher scores denote worse mental health. Scores on the PHQ-2 and GAD-2 were included as covariates in the analyses.

#### Intervention engagement.

Intervention use was automatically tracked by the SES NXT platform whenever a module was started or completed. A composite variable was constructed to reflect the total number of modules started or completed by each participant. This measure was used in secondary analyses to examine potential dose-response effects.

**Somatic complaints:** Somatic complaints were measured by asking whether the youth experienced headaches, stomach aches, or nausea. The item was rated on a 3-point scale (i.e., 0 = “not true”, 1 = “somewhat true”, and 2 = “certainly true”). This item is item 3 in the SDQ Emotional Symptoms subscale [60,61]. The SDQ is a widely used and validated screening instrument for child and adolescent mental health across clinical and population-based samples [60,61]. This item has also previously been used in a national cohort investigation of children’s well-being in Denmark [15].

**Sleep latency problems:** Sleep latency was assessed by asking whether the youth had had difficulties falling asleep during the last month. Participants were provided with the following response options: “never” (1), “a couple of times” (2), “almost every week” (3), “more than once a week” (4), and “almost every day” (5). This item was drawn from the Danish version of the Health Behaviour in School-aged Children (HBSC) study [62,63]. The HBSC questionnaire is an international, school-based survey with established validity for monitoring health behaviours and subjective health complaints among children and adolescents [62,63].

**Body size perceptions:** Body size perceptions were assessed by asking how the youth perceived their body size, with response options ranging from 1 (“much too thin”) to 5 (“much too fat”). For analysis, the variable was recoded such that the middle response option (i.e., “just right”) was coded as 0; responses “a little too thin” and “a little too fat” were coded 1; and “much too thin” and “much too fat” were coded as 2. Higher scores thus indicate more extreme body size perceptions. This item was also drawn from the Danish version of the HBSC study.

### Plan of analysis

To examine the effectiveness of the intervention, multilevel regressions were conducted to examine group differences in terms of somatic complaints (H1), sleep latency problems (H2), and body size perceptions (H3) at T3. These analyses were based on the intention-to-treat principle [64]. Missing data was handled through single imputation, executed in R, using the package “mice”; roughly 12% of the data was imputed. The imputation strategy was changed because of the relatively low amount of missing data and because the nesting of the data made it difficult to ensure stable and correctly specified multiple imputations [65,66]. All main analyses were conducted in SAS version 9.4.

We initially examined the distribution of responses to our outcome measures (i.e., somatic complaints, sleep latency problems, and body size perceptions) in terms of frequencies. We then examined whether those in the intervention statistically differed from those in the WL control group on the responses. We employed generalized estimating equations, with a multinomial response profile, as estimated in PROC GENMOD, with a cumulative logit link function (please see analysis code and results output files on https://osf.io/5bpva/?view_only=125977a3950a42a6bca77498c416f853). For all outcomes, we modeled the probability of more severe symptomology (i.e., probability of indicating more somatic complaints, more sleep latency problems, and more extreme body size perceptions). The first set of analyses examined differences between the two groups at T3, as per protocol. For these analyses, youth were nested within family.

Additional follow-up analyses were executed to examine whether there was a differential change in probabilities over time for the two groups, as reflected in a group-by-time interaction effect, and these analyses accounted for nesting of time within youth and family. We also examined whether, for the intervention group, responses differed at T3 based on the intervention age group (age groups 3–5, 6–8, 9–12, and 13–17 years) and number of modules engaged with in the intervention (i.e., a dose-response effect). These were tested in the same analysis.

For all analyses, we included potential covariates; these were the baseline scores for the outcome of interest (this variable was not included in the group*time analyses, as it was part of the outcome variable), youth participant gender and age, parental gender and age, parent educational level and income, and parental depression and anxiety scores as covariates (T3 scores in the main analyses, and time-varying scores in the group*time analyses). A *p*-value of less than 0.05 was used as the threshold for statistical significance, since this value was used as the acceptable risk of type I error in our sample size estimation.

#### Sensitivity analyses.

Given that the imputation strategy was changed from multiple to single imputation, we conducted a series of additional analyses to examine the robustness of the results. For one, all GEE analyses were conducted on the unimputed dataset as well, and the pattern of results was consistent with those based on the imputed data, with one exception (i.e., a dose-response effect was non-significant in the unimputed data, but significant in the imputed data). Table J in S1 Appendix depicts the percentage of missingness for the outcome variables and main covariates, by intervention and WL group, across time. Results tables for the analyses using the unimputed data can be found in Fig A and Tables K–S in S1 Appendix.

Additionally, likelihood-based mixed-effects ordinal models were explored (using SAS proc glimmix) but failed to converge due to sparse within-cluster information (i.e., family sizes were small, relative to a parameter-heavy model). Models that estimated the fixed effects only (i.e., models that omitted random and nesting effects) converged and yielded results that were substantively similar in pattern to those obtained by the reported GEE models. This applied to analyses conducted on both the imputed and the non-imputed data. Output files can be found https://osf.io/5bpva/?view_only=125977a3950a42a6bca77498c416f853.

## Supporting information

S1 Appendix This file contains Fig A and Tables A-T. (DOCX)
