# Supplementary material for: Effects of a digital psychosocial intervention on somatic complaints, sleep latency problems, and body size perceptions in youth after parental separation: A randomized controlled trial
Source: PLOS Digit Health. 2026 Jun 18;5(6):e0001460. doi: 10.1371/journal.pdig.0001460 (PMC13278405; doi:10.1371/journal.pdig.0001460)
Supplement: S1 Appendix — (DOCX) [file pdig.0001460.s001.docx]

**S1 Appendix. Supplemental materials for the manuscript.**

This file contains Fig A and Tables A-T.

**Table A.** *Marginal (unadjusted) predicted probabilities of responses to the three outcomes at T3, for intervention and WL control group separately*

|  | Intervention group | WL Control Group |
| --- | --- | --- |
| *Somatic Complaints: I often have headaches, stomach aches, or nausea* | | |
| 2. Certainly true | 0.08610 | 0.2831 |
| 1. Somewhat true | 0.3543 | 0.6970 |
| *Sleep Latency Problems: How often have you experienced having difficulty falling asleep during the past month?* | | |
| 5. Almost every day | 0.02325 | 0.09993 |
| 4. More than once per week | 0.1229 | 0.3953 |
| 3. Almost every week | 0.2615 | 0.6228 |
| 2. A couple of times | 0.8293 | 0.9577 |
| *Extreme Body Size Perceptions: Do you think {NAME} is:* | | |
| 2. Too thin/fat | 0.02049 | 0.05026 |
| 1. A bit too thin/fat | 0.1959 | 0.3814 |

Note. SAS does not provide probabilities for the reference group.

**Somatic complaints** *b* = 1.43, z = 9.54, *p* <.001, *OR* = 4.192

**Sleep latency problems** *b* = 1.54, z = 9.49, *p* <.001, *OR* = 4.664

**Body size perceptions** *b* = 0.928, z = 6.01, *p* <.001, *OR* = 2.530

**Table B.** *Marginal (covariate-adjusted) predicted probabilities of responses to the three outcomes at T3, for intervention and WL control group separately*

|  | Intervention group | WL Control Group |
| --- | --- | --- |
| *Somatic Complaints: I often have headaches, stomach aches, or nausea* | | |
| 2. Certainly true | 0.07 | 0.24 |
| 1. Somewhat true | 0.35 | 0.69 |
| *Sleep Latency Problems: How often have you experienced having difficulty falling asleep during the past month?* | | |
| 5. Almost every day | 0.02 | 0.06 |
| 4. More than once per week | 0.10 | 0.32 |
| 3. Almost every week | 0.26 | 0.58 |
| 2. A couple of times | 0.87 | 0.96 |
| *Extreme Body Size Perceptions: Do you think {NAME} is:* | | |
| 2. Too thin/fat | 0.01 | 0.01 |
| 1. A bit too thin/fat | 0.13 | 0.29 |

Note. SAS does not provide probabilities for the reference group.

**Table C.** *Estimated category-specific probabilities (in percent) for reporting somatic complaints, by group and time (multinomial GEE model)*

| **Study Group** | **Time** | **Certainly true (%)** | **Somewhat true (%)** |
| --- | --- | --- | --- |
| Control | 0 | 0.331 | 0.735 |
| Control | 1 | 0.303 | 0.708 |
| Control | 2 | 0.261 | 0.663 |
| Intervention | 0 | 0.342 | 0.744 |
| Intervention | 1 | 0.108 | 0.403 |
| Intervention | 2 | 0.086 | 0.344 |

**Note.** SAS does not provide probabilities for the reference group (i.e., “Not true”).

Estimated probabilities of reporting frequent somatic complaints (i.e., “certainly true”) decreased significantly in the intervention group from T1 (*M* = 34.2%) to T3 (*M* = 8.6%), whereas the control group remained relatively stable (33.1% at T1 to 26.1% at T3). A similar pattern was observed for moderate complaints (i.e., “somewhat true”).

**Table D.** *Estimated category-specific probabilities (in percent) of child sleep latency problems by group and wave from multinomial GEE model*

| **Group** | **Time** | **Almost**  **Daily** | **More Than**  **Once/Week** | **Almost**  **Weekly** | **A Few**  **Times** |
| --- | --- | --- | --- | --- | --- |
| Control | T1 | 0.095 | 0.407 | 0.650 | 0.947 |
| Control | T2 | 0.0856 | 0.381 | 0.624 | 0.941 |
| Control | T3 | 0.075 | 0.349 | 0.591 | 0.933 |
| Intervention | T1 | 0.107 | 0.442 | 0.681 | 0.954 |
| Intervention | T2 | 0.043 | 0.226 | 0.441 | 0.884 |
| Intervention | T3 | 0.024 | 0.138 | 0.302 | 0.806 |

**Note.** SAS does not provide probabilities for the reference group (i.e., “Never”).

Estimated probabilities of reporting frequent sleep latency problems (i.e., “almost daily”, “more than once/week”, and “almost weekly”) decreased significantly in the intervention group from T1 (for the “almost daily” category, from *M* = 10.7% at T1 to *M* = 2.4% at T3), whereas the control group remained relatively stable (9.5% at T1 to 7.5% at T3).

**Table E.** *Estimated category-specific probabilities (in percent) from a multinomial GEE model for body size perception category by group and time*

| **Group** | **Time** | **“much too thin”/**  **“much too fat”** | **“a little too thin”/**  **“a little too fat”** |
| --- | --- | --- | --- |
| Control | T1 | 0.045 | 0.359 |
| Control | T2 | 0.041 | 0.334 |
| Control | T3 | 0.046 | 0.363 |
| Intervention | T1 | 0.038 | 0.322 |
| Intervention | T2 | 0.031 | 0.274 |
| Intervention | T3 | 0.020 | 0.198 |

**Note.** SAS does not provide probabilities for the reference group (i.e., “Just right”).

As shown in the table above, the estimated probability of being classified as “a little too thin”/“a little too fat” declined in the intervention group from T1 (*M* = 32.2%) to T3 (*M* = 19.8%) but remained stable for the control group (35.9% at T1 to 36.3% at T3).

**Table F.** *Frequencies of Number of Modules Completed*

|  | Overall  (N = 449) | | Intervention age group  3-5 (N = 109) | | Intervention age group  6-8 (N = 115) | | Intervention age group  9-12 (N = 122) | | Intervention age group  13-17 (N = 103) | |
| --- | --- | --- | --- | --- | --- | --- | --- | --- | --- | --- |
| Modules | Frequency | Percent | Frequency | Percent | Frequency | Percent | Frequency | Percent | Frequency | Percent |
| 0 | 94 | 20.94 | 16 | 14.68 | 30 | 26.09 | 33 | 27.05 | 15 | 14.56 |
| 1 | 107 | 23.83 | 93 | 85.32 | 6 | 5.22 | 6 | 4.92 | 2 | 1.94 |
| 2 | 10 | 2.23 | . | . | 3 | 2.61 | 4 | 3.28 | 3 | 2.91 |
| 3 | 22 | 4.90 | . | . | 7 | 6.09 | 9 | 7.38 | 6 | 5.83 |
| 4 | 51 | 11.36 | . | . | 17 | 14.78 | 12 | 9.84 | 22 | 21.36 |
| 5 | 61 | 13.59 | . | . | 21 | 18.26 | 14 | 11.48 | 26 | 25.24 |
| 6 | 48 | 10.69 | . | . | 19 | 16.52 | 15 | 12.30 | 14 | 13.59 |
| 7 | 32 | 7.13 | . | . | 6 | 5.22 | 15 | 12.30 | 11 | 10.68 |
| 8 | 16 | 3.56 | . | . | 6 | 5.22 | 8 | 6.56 | 2 | 1.94 |
| 9 | 7 | 1.56 | . | . | . | . | 6 | 4.92 | 1 | 0.97 |
| 10 | 1 | 0.22 | . | . | . | . | . | . | 1 | 0.97 |

Note. For Intervention age group 3-5, the intervention consisted of 1 module that had four themes; for Intervention age group 6-8, the intervention consisted of 8 modules; for Intervention age group 9-12 and Intervention age group 13-17, the intervention consisted of 10 modules.

**Table G.** *Displaying the Frequencies of Completion of the Modules*

| **Theme** | Intervention age group  6-8 (N = 115) | | Intervention age group  9-12 (N = 122) | | Intervention age group  13-17 (N = 103) | |
| --- | --- | --- | --- | --- | --- | --- |
|  | Frequency | Percent | Frequency | Percent | Frequency | Percent |
| **Family Constellations** |  |  |  |  |  |  |
| The Bonus Family | 25 | 21.74 | 43 | 35.25 | 22 | 21.36 |
| **Practical Matters** |  |  |  |  |  |  |
| Living in Two Places | 56 | 48.70 | 48 | 39.34 | 47 | 45.63 |
| Packing Your Bag | 37 | 32.17 | 32 | 26.23 | 18 | 17.48 |
| **Emotional Aspects of Parental Divorce** | | | | | | |
| Understand Your Feelings | 74 | 64.35 | 55 | 45.08 | 51 | 49.51 |
| When It has Just Happened | 23 | 20.00 | 26 | 21.31 | 17 | 16.50 |
| Tell Your Story | . | . | 39 | 31.97 | 44 | 42.72 |
| **Agency** |  |  |  |  |  |  |
| Find an Important Adult | 60 | 52.17 | 46 | 37.70 | 47 | 46.60 |
| Learn To Say Yes and No | 63 | 54.78 | 62 | 50.82 | 61 | 60.19 |
| My Parents Are Not Getting Along | 71 | 61.74 | 45 | 36.89 | 63 | 61.17 |
| My Rights | . | . | 56 | 45.90 | 68 | 66.02 |

**Note.** Frequencies are not displayed for age group 3-5, as they completed a single module that had four themes.

**Table H.** *Depicting the Effect of Intervention Themes at 12-week Follow-up (T3) in the Intervention Group*

|  | Theme: Family Constellations | | | Theme: Practical Matters | | | Theme: Emotional Aspects of Parental Divorce | | | Theme: Agency | | | |
| --- | --- | --- | --- | --- | --- | --- | --- | --- | --- | --- | --- | --- | --- |
| Outcome Measure | *b* value | *z* value | *p* value | *b* value | *z* value | *p* value | *b* value | *z* value | *p* value | | *b* value | *z* value | *p* value |
| Somatic Complaints | 0.0630 | 0.22 | 0.8245 | -0.0965 | -0.61 | 0.5391 | 0.2306 | 1.43 | 0.1538 | | -0.1826 | -1.79 | 0.0731 |
| Sleep Latency Problems | 0.1483 | 0.49 | 0.6217 | -0.0491 | -0.32 | 0.7505 | -0.0605 | -0.43 | 0.6680 | | -0.1403 | -1.35 | 0.1783 |
| Body Size Problems | 0.0620 | 0.17 | 0.8647 | -0.0294 | -0.14 | 0.8912 | -0.4750 | -2.09 | 0.0363 | | -0.1633 | -1.28 | 0.2014 |
| **Note.** N_intervention_ = 449. T1 = Baseline, T3 = 12-week follow-up. The results (unstandardized estimate *b*, *z*-value and *p*-value) come from Analysis of GEE Parameter Estimates (WL vs. intervention group). These tests are standard output from SAS proc genmod; model / type3. Covariates (i.e., participants T1 score on the outcome, participants' gender and age, parental gender and age, educational level, income, parental mental health symptoms at T3, and parent- vs self-report) were included in the analysis. | | | | | | | | | | | | | |

**Table I.** *Examination of Potential Attrition Bias: Baseline Sociodemographic Variables, Child Well-being Indicators, and Parental Mental Health Predicting Attrition (i.e., completing only the T1 survey)*

| Source | *b* | Chi-Square | *p*-value | *OR* |
| --- | --- | --- | --- | --- |
| Group | **-.80** | **8.38** | **0.0038** | **.44** |
| Intervention age group | - | 4.68 | 0.1965 | -- |
| Somatic complaints | -.13 | 3.71 | 0.0540 | .88 |
| Sleep latency problems | **-.12** | **6.31** | **0.0120** | **.89** |
| Extreme body size perceptions | .01 | 0.01 | 0.9033 | 1.01 |
| Child gender | -.02 | 0.04 | 0.8487 | .98 |
| Child age | .09 | 2.39 | 0.1219 | 1.09 |
| Parent gender | -.48 | 2.32 | 0.1280 | .62 |
| Parent age | **.07** | **7.40** | **0.0065** | **1.07** |
| Parent income | **-.23** | **6.18** | **0.0129** | **.79** |
| Parent educational level | - | 0.88 | 0.6440 | - |
| Parent depression symptoms | .07 | 0.35 | 0.5532 | 1.07 |
| Parent anxiety symptoms | .04 | 0.19 | 0.6639 | 1.04 |

Note. *N* = 866. The unstandardized regression coefficient (*b*) comes from Analysis of GEE Parameter Estimates, while the test statistics (chi-square and p-value) come from a Type 3 GEE Analysis. These tests are standard output from SAS proc genmod; model / type3 dist = binomial (to obtain results for a logistic regression). The unstandardized regression coefficients are only provided for continuous and binary predictors. For Gender-variables, female is the reference group. For the Group variable, intervention group was the reference group. Bold font indicates statistical significance. Analyses were conducted on the imputed data set.

**Sensitivity analysis**

**Below are the results summary and tables for analyses conducted on the raw/unimputed data file. The file contained the 866 youth that were included in the main analyses; we analyzed all available data. The analyses mirrored those reported in the manuscript in terms of covariate inclusion and model specification. All output files can be found at** [**https://osf.io/wyu92**](https://osf.io/wyu92)**.**

**Fig A. Distribution of responses for somatic complaints, sleep latency problems, and body size perceptions across study groups and time points.** Bars show the percentage distribution of response categories for the waitlist (WL) control and intervention groups at baseline (T1), 4 weeks after enrollment (T2), and 12 weeks after enrollment (T3).


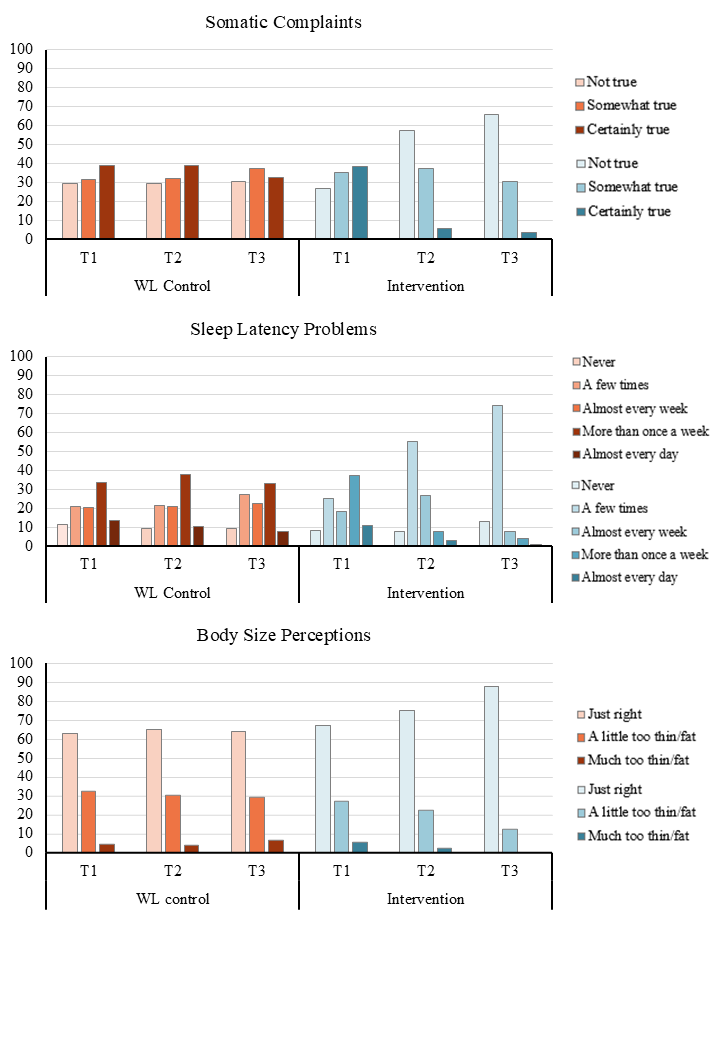


Note. Variable Ns for across study group and time, due to attrition

**Table J**. *Information on missingness (N/%) for all outcome variables and main demographic covariates, by group*

|  | WL control group | | | Intervention group | | |
| --- | --- | --- | --- | --- | --- | --- |
| *Variable* | *N* | *N Miss* | *Percent missing* | *N* | *N Miss* | *Percent missing* |
| Somatic Complaints - T1 | 416 | 1 | 0.24 | 448 | 1 | 0.22 |
| Somatic Complaints – T2 | 380 | 37 | 8.87 | 359 | 90 | 20.04 |
| Somatic Complaints – T3 | 368 | 49 | 11.75 | 343 | 106 | 23.61 |
| Sleep Latency Problems – T1 | 402 | 15 | 3.60 | 435 | 14 | 3.12 |
| Sleep Latency Problems – T2 | 380 | 37 | 8.87 | 345 | 104 | 23.16 |
| Sleep Latency Problems - T3 | 326 | 91 | 21.82 | 308 | 141 | 31.40 |
| Extreme Body Size Perceptions – T1 | 413 | 4 | 0.96 | 448 | 1 | 0.22 |
| Extreme Body Size Perceptions – T2 | 375 | 42 | 10.07 | 357 | 92 | 20.49 |
| Extreme Body Size Perceptions – T3 | 362 | 55 | 13.19 | 339 | 110 | 24.50 |
| Child gender | 415 | 2 | 0.48 | 448 | 1 | 0.22 |
| Child age | 417 | 0 | 0 | 449 | 0 | 0 |
| Parent gender | 208 | 15 | 6.73 | 227 | 17 | 6.97 |
| Parent age | 208 | 15 | 6.73 | 227 | 17 | 6.97 |
| Parent income | 208 | 15 | 6.73 | 227 | 17 | 6.97 |
| Parent educational level | 208 | 15 | 6.73 | 227 | 17 | 6.97 |

Note. N = number of people who completed the question; N miss = number of people for whom the value is missing; percent missing = the percentage of people for whom the value is missing.

**Table K.** *Marginal (unadjusted) predicted probabilities of responses to the three outcomes at T3, for intervention and WL control group separately*

|  | Intervention group | WL Control Group |
| --- | --- | --- |
| *Somatic Complaints: I often have headaches, stomach aches, or nausea (N = 711)* | | |
| 2. Certainly true | 0.07452 | 0.2975 |
| 1. Somewhat true | 0.3309 | 0.7223 |
| *Sleep Latency Problems: How often have you experienced having difficulty falling asleep during the past month? (N = 634)* | | |
| 5. Almost every day | 0.01167 | 0.07805 |
| 4. More than once per week | 0.08229 | 0.3913 |
| 3. Almost every week | 0.1834 | 0.6169 |
| 2. A couple of times | 0.8154 | 0.9694 |
| *Extreme Body Size Perceptions: Do you think {NAME} is: (N = 701)* | | |
| 2. Too thin/fat | 0.01370 | 0.05399 |
| 1. A bit too thin/fat | 0.1222 | 0.3638 |

Note. SAS does not provide probabilities for the reference group.

**Somatic complaints** *b* = 1.65, z = 10.00, *p* <.001, *OR* = 5.258

**Sleep latency problems** *b* = 1.97, z = 9.43, *p* <.001, *OR* = 7.168

**Body size perceptions** *b* = 1.41, z = 7.13, *p* <.001, *OR* = 4.109

**Table L.** *Tests of Effectiveness of the Intervention, As Defined by a Test of Group Differences at T3.*

| Outcome | N | Estimate | St. Error | *95% CI* | | *z* | *p* | *OR* |
| --- | --- | --- | --- | --- | --- | --- | --- | --- |
| Somatic complaints | 646 | 1.7608 | 0.2153 | 1.3389 | 2.1827 | 8.18 | <.0001 | 5.817 |
| Sleep latency problems | 570 | 2.0812 | 0.2690 | 1.5540 | 2.6085 | 7.74 | <.0001 | 8.014 |
| Body size perceptions | 639 | 2.0558 | 0.3751 | 1.3206 | 2.7909 | 5.48 | <.0001 | 7.813 |

**Table M.** *Marginal (covariate-adjusted) predicted probabilities of responses to the three outcomes at T3, for intervention and WL control group separately*

|  | Intervention group | WL Control Group |
| --- | --- | --- |
| *Somatic Complaints: I often have headaches, stomach aches, or nausea* | | |
| 2. Certainly true | 0.05253 | 0.2438 |
| 1. Somewhat true | 0.3263 | 0.7381 |
| *Sleep Latency Problems: How often have you experienced having difficulty falling asleep during the past month?* | | |
| 5. Almost every day | 0.004108 | 0.03200 |
| 4. More than once per week | 0.04138 | 0.2570 |
| 3. Almost every week | 0.1415 | 0.5691 |
| 2. A couple of times | 0.8764 | 0.9827 |
| *Extreme Body Size Perceptions: Do you think {NAME} is:* | | |
| 2. Too thin/fat | 0.000821 | 0.006376 |
| 1. A bit too thin/fat | 0.03278 | 0.2093 |

Note. SAS does not provide probabilities for the reference group.

**Table N.** *Tests of Effectiveness of the Intervention, As Defined by a Test of Group Differences Over Time, as well as Tests of the Effect of Intervention Age, Version and Intervention Use.*

| Outcome | Group x Time | | Intervention age group^a^ | | Modules^a^ | |
| --- | --- | --- | --- | --- | --- | --- |
|  | *Chi-square* | *p-value* | *Chi-square* | *p-value* | *Chi-square* | *p-value* |
| Somatic complaints | 82.52 | <.0001 | 6.11 | 0.1066 | 0.08 | 0.7734 |
| Sleep latency problems | 88.88 | <.0001 | 7.10 | 0.0687 | 1.77 | 0.1828 |
| Body size perceptions | 37.98 | <.0001 | 7.73 | 0.0520 | 1.99 | 0.1588 |

**Table O.** *Tests of Comparison Between the WL Control Group and Intervention Group at Each Time Point for Somatic Complaints, Sleep Latency Problems, and Body Size Perceptions.*

|  |  |  | Somatic complaints | | | Sleep latency problems | | | Body size perceptions | | |
| --- | --- | --- | --- | --- | --- | --- | --- | --- | --- | --- | --- |
| Comparison |  |  | Estimate | *p* | *OR* | Estimate | *p* | *OR* | Estimate | *p* | *OR* |
| Control group vs. | Intervention group | at T1 | -0.04010 | 0.7839 | 0.961 | -0.1510 | 0.3186 | 0.860 | 0.1665 | 0.2814 | 1.181 |
| Control group vs. | Intervention group | at T2 | 1.5377 | <.0001 | 4.654 | 1.1737 | <.0001 | 3.234 | 0.4519 | 0.0118 | 1.571 |
| Control group vs. | Intervention group | at T3 | 1.5957 | <.0001 | 4.932 | 1.5473 | <.0001 | 4.699 | 1.3057 | <.0001 | 3.690 |

**Table P.** *Estimated category-specific probabilities (in percent) for reporting somatic complaints, by group and time (multinomial GEE model)*

| **Study Group** | **Time** | **Certainly true (%)** | **Somewhat true (%)** |
| --- | --- | --- | --- |
| Control | 0 | 0.3341 | 0.7475 |
| Control | 1 | 0.3345 | 0.7478 |
| Control | 2 | 0.2799 | 0.6963 |
| Intervention | 0 | 0.3431 | 0.7550 |
| Intervention | 1 | 0.09746 | 0.3891 |
| Intervention | 2 | 0.07306 | 0.3174 |

**Note.** SAS does not provide probabilities for the reference group (i.e., “Not true”).

Estimated probabilities of reporting frequent somatic complaints (i.e., “certainly true”) decreased significantly in the intervention group from T1 (*M* = 34.3%) to T3 (*M* = 7.3%), whereas the control group remained relatively stable (33.4% at T1 to 28.0% at T3). A similar pattern was observed for moderate complaints (i.e., “somewhat true”).

**Table Q.** *Estimated category-specific probabilities (in percent) of child sleep latency problems by group and wave from multinomial GEE model*

| **Group** | **Time** | **Almost**  **Daily** | **More Than**  **Once/Week** | **Almost**  **Weekly** | **A Few**  **Times** |
| --- | --- | --- | --- | --- | --- |
| Control | T1 | 0.08267 | 0.4159 | 0.6695 | 0.9591 |
| Control | T2 | 0.08073 | 0.4096 | 0.6638 | 0.9580 |
| Control | T3 | 0.05946 | 0.3331 | 0.5870 | 0.9427 |
| Intervention | T1 | 0.09487 | 0.4530 | 0.7021 | 0.9646 |
| Intervention | T2 | 0.02644 | 0.1767 | 0.3791 | 0.8759 |
| Intervention | T3 | 0.01328 | 0.09609 | 0.2322 | 0.7777 |

**Note.** SAS does not provide probabilities for the reference group (i.e., “Never”).

Estimated probabilities of reporting frequent sleep latency problems (i.e., “almost daily”, “more than once/week”, and “almost weekly”) decreased significantly in the intervention group from T1 (for the “almost daily” category, from *M* = 9.5% at T1 to *M* = 1.3% at T3), whereas the control group remained relatively stable (8.3% at T1 to 5.9% at T3).

**Table R.** *Estimated category-specific probabilities (in percent) from a multinomial GEE model for body size perception category by group and time*

| **Group** | **Time** | **“much too thin”/**  **“much too fat”** | **“a little too thin”/**  **“a little too fat”** |
| --- | --- | --- | --- |
| Control | T1 | 0.04359 | 0.3447 |
| Control | T2 | 0.04043 | 0.3271 |
| Control | T3 | 0.04102 | 0.3305 |
| Intervention | T1 | 0.03715 | 0.3081 |
| Intervention | T2 | 0.02611 | 0.2363 |
| Intervention | T3 | 0.01146 | 0.1180 |

**Note.** SAS does not provide probabilities for the reference group (i.e., “Just right”).

As shown in the table above, the estimated probability of being classified as “a little too thin”/“a little too fat” declined in the intervention group from T1 (*M* = 30.8%) to T3 (*M* = 11.8%) but remained stable for the control group (34.5% at T1 to 33.1% at T3).

**Table S.** *Depicting the Effect of Intervention Themes at 12-week Follow-up (T3) in the Intervention Group*

|  | Theme: Family Constellations | | | Theme: Practical Matters | | | Theme: Emotional Aspects of Parental Divorce | | | Theme: Agency | | | |
| --- | --- | --- | --- | --- | --- | --- | --- | --- | --- | --- | --- | --- | --- |
| Outcome Measure | *b* value | *z* value | *p* value | *b* value | *z* value | *p* value | *b* value | *z* value | *p* value | | *b* value | *z* value | *p* value |
| Somatic Complaints | 0.1211 | 0.36 | 0.7160 | -0.1097 | -0.59 | 0.5567 | 0.1806 | 0.96 | 0.3374 | | -0.0357 | -0.29 | 0.7713 |
| Sleep Latency Problems | -0.3345 | -0.89 | 0.3709 | 0.1562 | 0.66 | 0.5108 | 0.2956 | 1.24 | 0.2151 | | 0.1290 | 0.82 | 0.4120 |
| Body Size Problems | -0.2411 | -0.46 | 0.6450 | -0.0249 | -0.07 | 0.9427 | -0.5886 | -1.72 | 0.0860 | | 0.0297 | 0.15 | 0.8840 |
| **Note.** T3 = 12-week follow-up. | | | | | | | | | | | | | |

**Table T.** *CONSORT checklist.*

|  | Section/topic | No | CONSORT 2025 checklist item description | Reported on page no. |
| --- | --- | --- | --- | --- |
|  | **Title and abstract** | | |  |
|  | Title and structured abstract | 1a | Identification as a randomised trial | 1 |
|  |  | 1b | Structured summary of the trial design, methods, results, and conclusions | 2 |
|  | **Open science** | | |  |
|  | Trial registration | 2 | Name of trial registry, identifying number (with URL) and date of registration | 27–28 |
|  | Protocol and statistical analysis plan | 3 | Where the trial protocol and statistical analysis plan can be accessed | 22 |
|  | Data sharing | 4 | Where and how the individual de-identified participant data (including data dictionary), statistical code and any other materials can be accessed | 22 |
|  | Funding and conflicts of interest | 5a | Sources of funding and other support (eg, supply of drugs), and role of funders in the design, conduct, analysis and reporting of the trial | Online form |
|  |  | 5b | Financial and other conflicts of interest of the manuscript authors | Online form |
|  | **Introduction** | | |  |
|  | Background and rationale | 6 | Scientific background and rationale | 4–6 |
|  | Objectives | 7 | Specific objectives related to benefits and harms | 6 |
|  | **Methods** | | |  |
|  | Patient and public involvement | 8 | Details of patient or public involvement in the design, conduct and reporting of the trial | 30 |
|  | Trial design | 9 | Description of trial design including type of trial (eg, parallel group, crossover), allocation ratio, and framework (eg, superiority, equivalence, non-inferiority, exploratory) | 28–29 |
|  | Changes to trial protocol | 10 | Important changes to the trial after it commenced including any outcomes or analyses that were not prespecified, with reason | 22, 36–37 |
|  | Trial setting | 11 | Settings (eg, community, hospital) and locations (eg, countries, sites) where the trial was conducted | 23, 28–29 |
|  | Eligibility criteria | 12a | Eligibility criteria for participants | 23 |
|  |  | 12b | If applicable, eligibility criteria for sites and for individuals delivering the interventions (eg, surgeons, physiotherapists) | N/A |
|  | Intervention and comparator | 13 | Intervention and comparator with sufficient details to allow replication. If relevant, where additional materials describing the intervention and comparator (eg, intervention manual) can be accessed | 28–33; ref. [32] |
|  | Outcomes | 14 | Prespecified primary and secondary outcomes, including the specific measurement variable (eg, systolic blood pressure), analysis metric (eg, change from baseline, final value, time to event), method of aggregation (eg, median, proportion), and time point for each outcome | 28, 35–36 |
|  | Harms | 15 | How harms were defined and assessed (eg, systematically, non-systematically) | N/A |
|  | Sample size | 16a | How sample size was determined, including all assumptions supporting the sample size calculation | 22–23 |
|  |  | 16b | Explanation of any interim analyses and stopping guidelines | N/A |
|  | Randomisation: |  |  |  |
|  | Sequence generation | 17a | Who generated the random allocation sequence and the method used | 29 |
|  |  | 17b | Type of randomisation and details of any restriction (eg, stratification, blocking and block size) | 29 |
|  |  |  |  | **Reported on page no.** |
|  | Allocation concealment mechanism | 18 | Mechanism used to implement the random allocation sequence (eg, central computer/telephone; sequentially numbered, opaque, sealed containers), describing any steps to conceal the sequence until interventions were assigned | 29 |
|  | Implementation | 19 | Whether the personnel who enrolled and those who assigned participants to the interventions had access to the random allocation sequence | 29 |
|  | Blinding | 20a | Who was blinded after assignment to interventions (eg, participants, care providers, outcome assessors, data analysts) | 29 |
|  |  | 20b | If blinded, how blinding was achieved and description of the similarity of interventions | N/A |
|  | Statistical methods | 21a | Statistical methods used to compare groups for primary and secondary outcomes, including harms | 36–37 |
|  |  | 21b | Definition of who is included in each analysis (eg, all randomised participants), and in which group | 23, 36 |
|  |  | 21c | How missing data were handled in the analysis | 22, 36–37 |
|  |  | 21d | Methods for any additional analyses (eg, subgroup and sensitivity analyses), distinguishing prespecified from post hoc | 36–37; S1 Appendix |
|  | **Results** | | |  |
|  | Participant flow, including flow diagram | 22a | For each group, the numbers of participants who were randomly assigned, received intended intervention, and were analysed for the primary outcome | 23–24 |
|  |  | 22b | For each group, losses and exclusions after randomisation, together with reasons | 23–24 |
|  | Recruitment | 23a | Dates defining the periods of recruitment and follow-up for outcomes of benefits and harms | 23 |
|  |  | 23b | If relevant, why the trial ended or was stopped | N/A |
|  | Intervention and comparator delivery | 24a | Intervention and comparator as they were actually administered (eg, where appropriate, who delivered the intervention/comparator, how participants adhered, whether they were delivered as intended (fidelity)) | 14, 29–33; S1 Appendix |
|  |  | 24b | Concomitant care received during the trial for each group | N/A |
|  | Baseline data | 25 | A table showing baseline demographic and clinical characteristics for each group | 25–27 |
|  | Numbers analysed,  outcomes and estimation | 26 | For each primary and secondary outcome, by group:  ● the number of participants included in the analysis  ● the number of participants with available data at the outcome time point  ● result for each group, and the estimated effect size and its precision (such as 95% confidence interval)  ● for binary outcomes, presentation of both absolute and relative effect size | 6–13, 23–24; S1 Appendix |
|  | Harms | 27 | All harms or unintended events in each group | N/A |
|  | Ancillary analyses | 28 | Any other analyses performed, including subgroup and sensitivity analyses, distinguishing pre-specified from post hoc | 10–14, 37; S1 Appendix |
|  | **Discussion** | | |  |
|  | Interpretation | 29 | Interpretation consistent with results, balancing benefits and harms, and considering other relevant evidence | 15–19 |
|  | Limitations | 30 | Trial limitations, addressing sources of potential bias, imprecision, generalisability, and, if relevant, multiplicity of analyses | 19–21 |

Citation: Hopewell S, Chan AW, Collins GS, Hróbjartsson A, Moher D, Schulz KF, et al. CONSORT 2025 Statement: updated guideline for reporting randomised trials. BMJ. 2025; 388:e081123. <https://dx.doi.org/10.1136/bmj-2024-081123> © 2025 Hopewell et al. This is an Open Access article distributed under the terms of the Creative Commons Attribution License (<https://creativecommons.org/licenses/by/4.0/>), which permits unrestricted use, distribution, and reproduction in any medium, provided the original work is properly cited.

*We strongly recommend reading this statement in conjunction with the CONSORT 2025 Explanation and Elaboration and/or the CONSORT 2025 Expanded Checklist for important clarifications on all the items. We also recommend reading relevant CONSORT extensions. See [www.consort-spirit.org](http://www.consort-spirit.org).
